# Distinct Epidemiology and Temporal Trends of Multidrug-Resistant Organisms in a Newly Established Hospital: A Five-Year Surveillance Study

**DOI:** 10.3390/antibiotics15090834

**Published:** 2026-08-27

**Authors:** Xiaoju Ma, Zhanjie Li, Zhaofan Luo, Yan Lu, Mingming Chen, Runhan Huang, Xinhai Zhao, Li Du, Huiping Huang, Youpeng Chen

**Affiliations:** 1 Department of Medical Affairs, The Seventh Affiliated Hospital, Sun Yat-sen University, Shenzhen 518107, China; maxiaoju@sysush.com (X.M.);; 2 Department of Epidemiology and Health Statistics, School of Public Health, Fudan University, Shanghai 200032, China; 3 Department of Infection Control, The First Affiliated Hospital with Nanjing Medical University, Nanjing 210029, China; 4 Department of Clinical Medical Laboratory, The Seventh Affiliated Hospital, Sun Yat-sen University, Shenzhen 518107, China; 5 Department of Patient Experience, The Seventh Affiliated Hospital, Sun Yat-sen University, Shenzhen 518107, China; 6 Department of Infectious Diseases, The Seventh Affiliated Hospital, Sun Yat-sen University, Shenzhen 518107, China

**Keywords:** multidrug-resistant organisms, antimicrobial resistance, newly established hospital, carbapenem-resistant *Pseudomonas aeruginosa*, hospital epidemiology, infection prevention and control

## Abstract

**Background:** Antimicrobial resistance is a major global health challenge, with multidrug-resistant organisms (MDROs) emerging faster than new antimicrobial agents are being developed. The epidemiology of MDROs varies substantially across healthcare settings; however, newly established hospitals remain understudied. This study aimed to characterise the epidemiology and temporal trends of MDROs in a newly established tertiary hospital. **Methods:** A five-year retrospective longitudinal surveillance study was conducted from 2019 to 2023 in a newly established tertiary hospital in Southern China. Clinical isolates were collected from intensive care unit (ICU) and non-ICU wards. Nine major MDROs were investigated, including extended-spectrum β-lactamase-producing *Klebsiella pneumoniae* (ESBL-KP) and *Escherichia coli* (ESBL-ECO); carbapenem-resistant *Klebsiella pneumoniae* (CRKP), *Escherichia coli* (CRECO), *Acinetobacter baumannii* (CRAB), and *Pseudomonas aeruginosa* (CRPA); methicillin-resistant *Staphylococcus aureus* (MRSA); and vancomycin-resistant *Enterococcus faecium* (VREfm) and *Enterococcus faecalis* (VREfa). **Results:** A total of 1533 MDRO isolates were identified during the study period. The predominant pathogens were ESBL-ECO (48.1%), ESBL-KP (16.4%), MRSA (11.4%), and CRPA (11.1%). Most isolates were recovered from non-ICU wards (85.4%), and more than half (51.5%) were community-onset. Isolation rates of ESBL-ECO, CRECO, CRAB, and CRPA were significantly higher in the ICU than in non-ICU wards (all *p* < 0.05). The incidence density rate of hospital-acquired infections caused by ESBL-KP, CRAB, and CRPA was also significantly higher in the ICU (all *p* < 0.05). Longitudinal analysis revealed divergent trends among MDROs. While most MDRO isolation rates remained below provincial and national benchmark levels, CRKP and CRAB showed significant increasing trends. CRPA exhibited a distinct pattern, increasing rapidly during the early years after hospital opening, exceeding both provincial and national benchmarks by the second year and remaining elevated thereafter. **Conclusions:** MDRO epidemiology during the first five complete calendar years after hospital opening was characterised by a predominance of ESBL-ECO, ESBL-KP, MRSA, and CRPA, together with substantial non-ICU and community-onset burdens and heterogeneous temporal patterns across organisms. CRPA increased rapidly and remained above the available external benchmark thereafter. These findings highlight the need for hospital-wide, organism-specific MDRO surveillance, while maintaining intensified infection prevention and control in ICUs and continued vigilance for carbapenem-resistant pathogens, particularly CRPA.

## 1. Background

Antimicrobial resistance (AMR) is one of the most pressing global health challenges of the 21st century, with multidrug resistance emerging as a major threat worldwide [1]. The emergence and spread of multidrug-resistant organisms (MDROs) are outpacing the development of new antimicrobials, resulting in an increasing number of infections for which effective treatment options are limited [2]. The epidemiology of MDROs varies considerably across regions, hospitals and clinical settings [3]. As healthcare facilities are major reservoirs for the selection, amplification, and dissemination of MDROs [4], understanding their local epidemiology is essential for developing effective surveillance and infection prevention strategies.

Previous studies have suggested that local MDRO epidemiology may begin to evolve soon after a newly established hospital commences clinical operations. In a newly opened intensive care unit, carbapenem-resistant *Klebsiella pneumoniae* (CRKP) was first detected in the environment 10 weeks after commissioning [5], while progressive environmental contamination and accumulation of carbapenem-resistant Gram-negative bacteria have been reported in newly commissioned wards [6,7]. Taken together, these findings indicate that changes in local MDRO patterns may occur progressively and at different times for different organisms, highlighting the value of longitudinal surveillance during the initial years of hospital operation.

However, existing research in newly established hospitals has focused primarily on the environmental detection and accumulation of selected MDROs [5,6,7]. Few studies have systematically investigated how hospital-wide MDRO epidemiology evolves during the initial years after hospital opening. The present study therefore aimed to characterise the epidemiological profile, temporal trends, ward distribution and onset classification of nine major MDROs during the first five complete calendar years after the opening of a newly established tertiary teaching hospital in Southern China; to contextualise local isolation rates using available provincial and national surveillance data; and to describe the incidence and temporal trends of hospital-acquired MDRO infections.

## 2. Results

### 2.1. Epidemiological Profile of MDRO Isolates

A total of 1533 non-duplicate MDRO isolates were obtained from clinical specimens during the five-year surveillance period (2019–2023). Among 5216 included bacterial isolates, urine (*n* = 1630, 31.2%), respiratory tract specimens (*n* = 1150, 22.0%) and blood (*n* = 439, 8.4%) were the most frequent sources. Among the 1533 MDRO isolates, urine (*n* = 486, 31.7%), respiratory tract specimens (*n* = 406, 26.5%) and blood (*n* = 136, 8.9%) predominated (Table S1). Carbapenem-resistant *Acinetobacter baumannii* (CRAB) and *Pseudomonas aeruginosa* (CRPA) were recovered mainly from respiratory tract specimens (89.7% and 66.5%, respectively), whereas β-lactamase-producing *Escherichia coli* (ESBL-ECO) was recovered most frequently from urine (49.3%) (Table S2).

**Figure 1:**
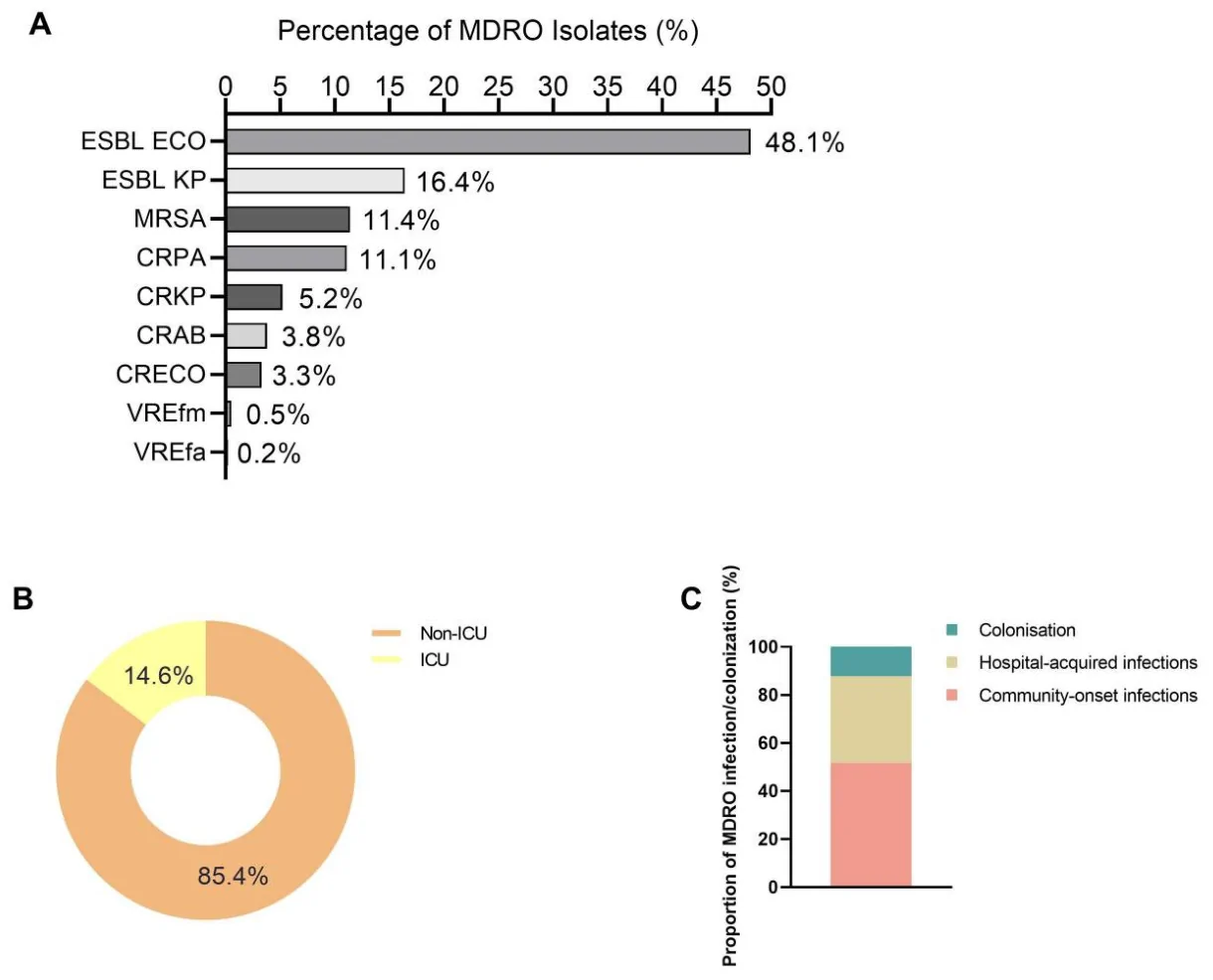
Epidemiological profile of MDRO isolates in the newly established hospital during 2019–2023. (**A**) Relative proportion of the nine targeted MDROs. (**B**) Ward-based distribution of all MDRO isolates. (**C**) Distribution of MDRO isolates according to clinical classification: colonisation, community-onset infection, and hospital-acquired infection. Abbreviations: ESBL, extended-spectrum β-lactamase-producing; MRSA, methicillin-resistant *Staphylococcus aureus*; CR, carbapenem-resistant; ECO, *Escherichia coli*; KP, *Klebsiella pneumoniae*; PA, *Pseudomonas aeruginosa*; AB, *Acinetobacter baumannii*; VREfa, vancomycin-resistant *Enterococcus faecalis*; VREfm, vancomycin-resistant *Enterococcus faecium*.

The overall epidemiological profile of MDROs was dominated by ESBL-ECO (48.1%), β-lactamase-producing *Klebsiella pneumoniae* (ESBL-KP) (16.4%), methicillin-resistant *Staphylococcus aureus* (MRSA) (11.4%), CRPA (11.1%), and CRKP (5.2%) (Figure 1A). Most MDRO isolates (85.4%) were identified in non-ICU wards (Figure 1B), while more than half (51.5%) were classified as community-onset infections (Figure 1C).

Ward-level analysis showed that the isolation rates of ESBL-ECO, carbapenem-resistant *Escherichia coli* (CRECO), CRAB, and CRPA were significantly higher in the ICU than in non-ICU wards (52.4% vs. 39.3%, 8.9% vs. 2.3%, 32.1% vs. 13.6%, and 44.3% vs. 24.5%, respectively; all *p* < 0.05) (Table 1).

### 2.2. Temporal Trends and External Benchmarking of MDRO Isolation Rates

**Figure 2:**
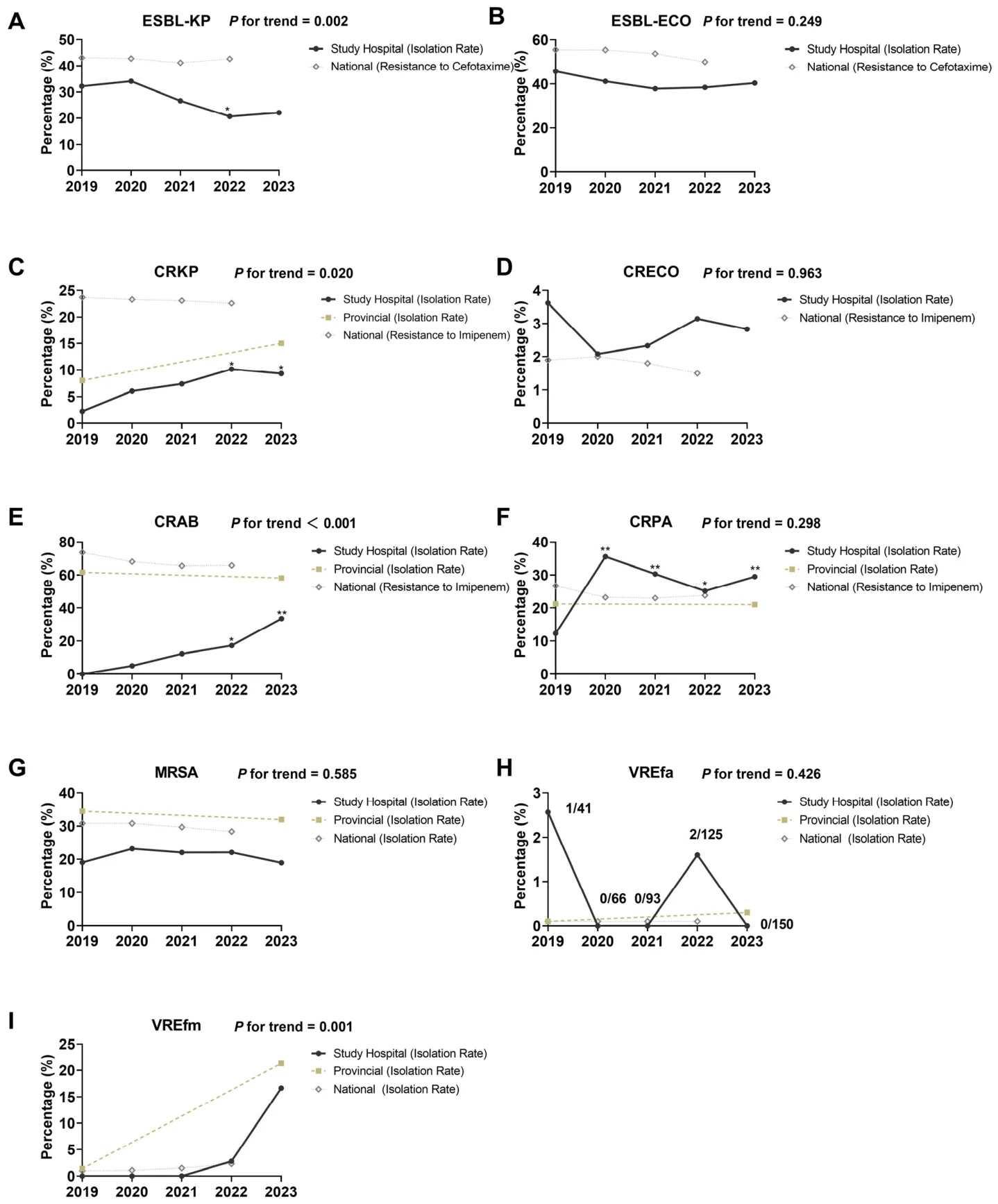
Temporal trends in isolation rates of key MDROs and comparison with provincial and national benchmarks (2019–2023). For each panel, the solid black line with circles represents the annual isolation rate (%) observed in the study hospital. The dashed green line indicates the corresponding provincial benchmark levels (reference isolation rate), based on surveillance data reported for 2019 and 2023 (data for the intervening years were not available). The dotted grey line represents national benchmark levels (reference isolation rate or resistance rates) derived from the China Antimicrobial Surveillance Network (CHINET) for the period 2019–2022. For ESBL-producing *E. coli* and *K. pneumoniae*, national resistance rates to cefotaxime were used; for carbapenem-resistant *K. pneumoniae*, *A. baumannii* and *P. aeruginosa*, resistance rates to imipenem were used. Data for VREfa are presented as *n*/N (number of resistant isolates/total isolates tested) owing to the limited number of positive cases detected during the study period. A significant temporal trend within the study hospital is indicated by *p* for trend < 0.05. * *p* < 0.05 or ** *p* < 0.01 compared with 2019. Abbreviations as in Figure 1.

Longitudinal analysis revealed divergent trends for different MDROs when contextualized with external surveillance data (Figure 2). The isolation rates of ESBL-KP and ESBL-ECO remained below the corresponding national benchmark levels during 2019–2022, for which national surveillance data were available (ESBL-KP: local 32.2% to 20.7% vs. national 43.1% to 42.7%; ESBL-ECO: local 45.7% to 38.4% vs. national 55.5% to 50.0%) (Figure 2A,B). The ESBL-KP rate exhibited a significant declining trend from 32.2% to 22.1% (*p* for trend = 0.002) (Figure 2A).

Among carbapenem-resistant organisms, increasing trends were observed for CRKP and CRAB, whereas the CRECO isolation rate remained relatively stable. CRKP and CRAB showed year-on-year increases, reaching 9.3% and 33.7% in 2023, respectively (both *p* for trend < 0.05). These rates remained below the corresponding 2023 provincial benchmarks of 15.0% and 58.2%, respectively (Figure 2C,E). In contrast, the CRECO isolation rate remained stable (2.8–3.6%) but consistently exceeded the corresponding national benchmark levels during 2019–2022, for which national surveillance data from the China Antimicrobial Surveillance Network (CHINET) were available (1.5–1.9%) (Figure 2D). Notably, CRPA exhibited a distinct pattern. The isolation rate of CRPA increased sharply from 12.3% in 2019 to 35.6% in 2020 and remained elevated at 29.5–30.3% during 2021–2023. During 2020–2022, the local rates exceeded the corresponding national benchmarks (23.2–23.8%), and the 2023 rate (29.5%) remained above the provincial benchmark (21.0%) (Figure 2F).

The isolation rate of MRSA remained stable throughout the period, ranging from 19.1% to 21.0%, and was lower than the available provincial benchmarks (34.4% in 2019 and 31.9% in 2023) and the national benchmarks (30.8% in 2019 to 28.4% in 2022) (Figure 2G). The number of isolated cases of VREfa was small, corresponding to a consistently low isolation rate (Figure 2H). The VREfm isolation rate increased significantly over the study period (*p* for trend = 0.001). Compared with the available provincial benchmarks, the local rate was lower in 2019 but reached a comparable level by 2023 (16.7% vs. 16.2%); it also exceeded the corresponding national benchmark of 2.3% in 2022 (Figure 2I).

### 2.3. Hospital-Acquired MDRO Incidence and Temporal Trends

Focusing on MDRO hospital-acquired infections, 554 episodes were identified, corresponding to an overall incidence density rate of 0.64 per 1000 patient days (Table 2). The incidence density rate of hospital-acquired MDRO infections was significantly higher in the ICU than in non-ICU wards (Table 2). In the ICU, the hospital-acquired incidence density rates of ESBL-KP, CRAB, and CRPA were higher than those in non-ICU wards (all *p* < 0.05).

**Figure 3:**
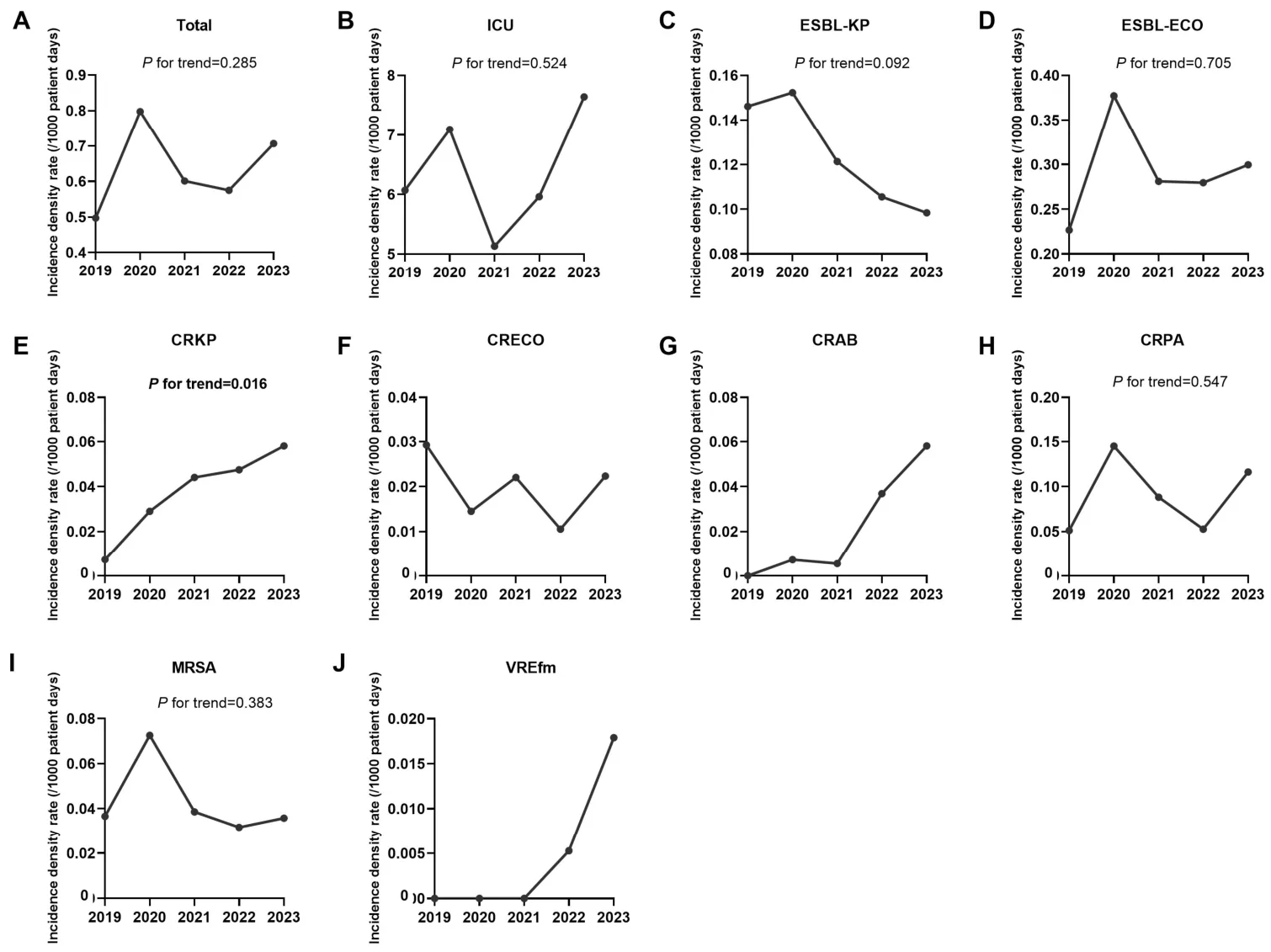
Temporal trends in the incidence density rate of hospital-acquired MDRO infections, 2019–2023. (**A**) Overall hospital-acquired MDRO infections. (**B**) Hospital-acquired MDRO infections in the ICU. (**C**) ESBL-KP. (**D**) ESBL-ECO. (**E**) CRKP. (**F**) CRECO. (**G**) CRAB. (**H**) CRPA. (**I**) MRSA. (**J**) VREfm. Statistically significant temporal trends are indicated by *p* for trend < 0.05. Trend analysis could not be performed for panels F, G and J because of the low number of events. Abbreviations as in Figure 2.

Over the five-year study period, the overall incidence density rate of hospital-acquired MDRO infections remained stable in the entire hospital and in the ICU (Figure 3A,B). A significant increasing trend was observed for CRKP-associated infections (*p* for trend = 0.016) (Figure 3E). No statistically significant temporal trends were observed for ESBL-KP, ESBL-ECO, CRPA, and MRSA-associated infections (Figure 3C,D,H,J). Trend analysis could not be performed for CRECO, CRAB, and VREfm due to low annual event numbers (Figure 3F,G,I).

### 2.4. Infection-Site Distribution and Clinical Characteristics and Outcomes

Among 1344 confirmed MDRO-associated infection episodes, urinary tract infection (431, 32.1%), lower respiratory tract infection or pneumonia (371, 27.6%), intra-abdominal infection (150, 11.2%) and bloodstream infection (136, 10.1%) were the most common infection types. Urinary tract infections were more frequent among community-onset infections (37.7% vs. 24.0%, *p* < 0.001), whereas bloodstream infections were more frequent among hospital-acquired infections (15.7% vs. 6.2%, *p* < 0.001). Hospital-acquired device-associated infections comprised 14 cases of ventilator-associated pneumonia, 56 cases of central line-associated bloodstream infection, and 48 cases of catheter-associated urinary tract infection (Table S3).

Among 1196 unique patients with confirmed MDRO-associated infections, patient-level analyses were performed to describe the clinical burden associated with different infection-site categories. Overall, 77 (6.4%) died in hospital, 297 (24.8%) were admitted to the ICU during the index hospitalisation, and the median length of hospital stay was 20 days (IQR, 10–37). Descriptively, the highest in-hospital mortality was observed among patients with ventilator-associated pneumonia (42.9%), followed by those with central line-associated bloodstream infection (18.9%). These subgroups also had longer median hospital stays, at 61 days (IQR, 28.8–127) and 43 days (IQR, 29–73), respectively. Detailed patient-level findings across infection-site categories and hospital-acquired device-associated infection subgroups are presented in Table S4.

### 2.5. Antimicrobial Consumption and Infection Prevention Compliance

Hospital-wide antimicrobial use intensity decreased from 39.61 DDDs per 100 bed-days in 2019 to 33.93 in 2021, before increasing modestly to 36.49 in 2022 and 37.12 in 2023.

Routine audits of subsets of MDRO cases showed variation in compliance with selected infection prevention measures across years (Table S5). Isolation signage remained above 90% throughout the study period, and environmental cleaning and disinfection improved after 2019. In contrast, compliance with personal protective equipment use declined in 2022–2023, while healthcare waste management also showed year-to-year variation.

## 3. Discussion

This study characterised MDRO epidemiology during the first five complete calendar years after a tertiary hospital opened. The MDRO profile was dominated by ESBL-ECO, ESBL-KP, MRSA, and CRPA. When contextualised against the available provincial and national surveillance benchmarks, the isolation rates of ESBL-ECO, ESBL-KP, and MRSA were generally lower, whereas CRPA showed a marked increase in 2020 and subsequently remained above the available corresponding benchmarks. This organism-specific profile also differs from the emphasis of previous reports from established tertiary hospitals, which have frequently focused on carbapenem-resistant organisms [8,9,10]. Our hospital showed lower isolation rates of ESBL-ECO, ESBL-KP, and MRSA than available external benchmarks, whereas CRPA exhibited an early increase followed by persistently elevated isolation rates. These organism-specific deviations from external benchmarks indicate that the local MDRO profile did not simply mirror broader surveillance trends and highlight the epidemiological value of external benchmarking for identifying locally distinct resistance patterns. By extending previous environmental observations to hospital-wide clinical surveillance, this study provides a broader characterisation of the MDRO profile in a newly established hospital.

The ICU had higher isolation rates for ESBL-ECO, CRECO, CRAB, and CRPA and higher incidence densities of hospital-acquired ESBL-KP, CRAB and CRPA infection, consistent with the recognised concentration of MDRO risk in critical care [11,12,13,14]. Nevertheless, 85.4% of all MDRO isolates were recovered from non-ICU wards, which may partly reflect the substantially larger bed capacity and patient population outside the ICU. Previous studies from established tertiary hospitals in China reported a stronger ICU concentration of CRAB, CRPA, and CRKP [11,15], whereas in our study, the absolute numbers of isolates of these organisms were higher in non-ICU wards. Although our hospital opened with the core clinical services and infrastructure of a tertiary general hospital in place, its shorter operational history distinguishes it from long-established institutions. Differences in operational history, together with variations in patient populations, clinical service structure, referral patterns, antimicrobial exposure, and local MDRO epidemiology may have contributed to the observed differences in ward distribution. Thus, ICU-focused surveillance alone may not fully capture the hospital-wide MDRO burden, although intensified ICU-specific measures remain necessary.

Temporal patterns varied considerably across organisms. ESBL-KP declined over the study period, whereas ESBL-ECO and MRSA remained relatively stable and below the available national benchmarks. Enhanced infection prevention and control (IPC) measures during the COVID-19 pandemic [16] and standard precautions implementation [17,18] may have contributed to these patterns. In contrast, carbapenem-resistant organisms showed distinct trajectories. The increase in CRKP appeared to parallel broader regional trends [10], likely influenced by regional epidemic trends, antimicrobial consumption, and resistance mechanisms [3,10,19]. The recognised environmental persistence of CRAB and its ability to survive under hospital conditions may provide biologically plausible explanations for its increasing trend [20,21,22]. Similarly, regional endemic pressure may have contributed to the gradual increase in VREfm observed in this study [10]. The COVID-19 pandemic overlapped with most of the study period and may have affected patient case mix, microbiological sampling, antimicrobial use, and infection prevention practices. Nevertheless, the divergent trajectories among organisms indicate that the observed patterns cannot be explained solely by a uniform pandemic-related effect. Hospital-wide antimicrobial consumption showed a modest decline during the first half of the study period, followed by a gradual increase thereafter. However, this aggregate pattern did not consistently correspond with organism-specific MDRO trajectories; for example, CRKP and CRAB increased despite the initial decline in overall antimicrobial consumption, whereas ESBL-KP decreased. These findings suggest that overall antimicrobial consumption alone was insufficient to explain the heterogeneous evolution of MDROs. However, antimicrobial-class-specific and ward-level consumption data were unavailable, preventing further assessment of these relationships. Notably, CRPA exhibited a sharp increase during the first two years of operation, surpassing stable provincial and national benchmarks and remaining persistently elevated thereafter; this pattern was not observed for other pathogens. This early, sustained elevation, combined with the finding that 74.7% of CRPA isolates were recovered from non-ICU wards, raises concern about possible hospital-wide dissemination and highlights the need for further molecular and environmental investigations [14,23,24]. Taken together, these findings highlight the value of organism-specific, hospital-wide longitudinal surveillance from the beginning of hospital operations, with particular attention to sustained high CRPA isolation rates.

Another notable finding is the dominant and increasing role of community-onset infections, which constituted over half (51.5%) of all MDRO isolates, a pattern consistent with other settings [25]. This suggests that a substantial proportion of MDROs were already present at the time of admission. Hospital-acquired infections accounted for 36.1% of all MDRO isolates, and the overall hospital-acquired burden remained relatively stable during the study period. Although a causal relationship cannot be established, this pattern was observed alongside sustained implementation of hospital-wide IPC measures, including consistently high compliance with isolation signage and improved compliance with environmental cleaning and disinfection [8,26], as well as a decreasing trend in healthcare-acquired ESBL-KP infections. These findings suggest that IPC strategies in newly established hospitals should address both community-onset and healthcare-associated MDROs. Internally, this requires sustained implementation of standard precautions and antimicrobial stewardship across all wards, while maintaining particular vigilance for pathogens showing sustained high isolation rates, especially CRPA. Furthermore, the increase in healthcare-associated CRKP infections, aligned with regional trends [10], underscores the need to incorporate regional surveillance intelligence into IPC planning.

Patient-level analyses further highlighted the clinical burden associated with MDRO-associated infections in this newly established hospital. Overall, 6.4% of patients with confirmed MDRO-associated infections died during hospitalisation, and the highest descriptive mortality and longest stays were observed among patients with ventilator-associated pneumonia and central line-associated bloodstream infection. These findings are consistent with previous evidence indicating that infections caused by antimicrobial-resistant organisms are associated with substantial clinical burden, including increased mortality and prolonged hospitalisation [27,28]. Taken together, these observations further demonstrate that MDRO surveillance should consider not only organism distribution and temporal trends but also the clinical burden associated with specific infection types.

This study has several limitations. First, its single-center design may limit the generalizability of the findings to other hospitals, particularly newly established institutions with different patient populations, service profiles, or infection prevention practices. Second, reliance on clinical culture-based detection without routine active screening for asymptomatic colonisation may have underestimated the true MDRO burden. Third, molecular typing and resistance-gene characterisation were not performed; therefore, clonal expansion, transmission pathways, specific resistance determinants, and carbapenemase types could not be determined. Fourth, provincial benchmark data were available only for 2019 and 2023, limiting year-by-year contextual comparisons. In addition, local isolation rates were not directly comparable with the multicentre resistance rates used for external benchmarking. Finally, only aggregate annual hospital-wide antimicrobial consumption data and selected infection prevention compliance data were available. The absence of antimicrobial-class-specific and ward-level antimicrobial consumption data, together with the ecological nature of these analyses, precluded causal assessment of the relationships between antimicrobial use, infection prevention measures, and organism-specific MDRO trends. In addition, as this study was designed primarily as a hospital-wide surveillance analysis, detailed individual-level exposure factors were not systematically assessed. Therefore, the observed clinical outcomes should be interpreted as descriptive findings, and causal relationships between MDRO-associated infections and clinical outcomes cannot be established because of potential confounding by disease severity and other patient-level factors.

In conclusion, this five-year longitudinal surveillance study characterised MDRO epidemiology during the first five complete calendar years after the opening of a newly established hospital. ESBL-producing Enterobacterales, MRSA, and CRPA predominated, with substantial non-ICU and community-onset burdens. Different MDRO categories showed heterogeneous temporal patterns, with CRPA demonstrating an early increase and persistently elevated isolation rates thereafter. Device-associated infections, particularly ventilator-associated pneumonia and central line-associated bloodstream infection, showed substantial descriptive clinical burden. These findings support hospital-wide, organism-specific surveillance, sustained attention to non-ICU wards and community-onset infections, intensified infection prevention and control in ICUs, and continued vigilance for CRPA. Future multicentre studies incorporating patient-level clinical data, genomic analyses, environmental surveillance, and antimicrobial-class-specific consumption data are needed to further elucidate the determinants and mechanisms underlying MDRO evolution in newly established hospitals.

## 4. Materials and Methods

### 4.1. Study Design and Setting

A five-year retrospective longitudinal surveillance study was conducted at the Seventh Affiliated Hospital, Sun Yat-sen University, a newly established 800-bed tertiary Grade A teaching hospital in Shenzhen, Guangdong, China. The hospital commenced clinical operations in May 2018 and serves both local residents and patients referred from neighbouring cities. It was established and opened as a tertiary general hospital, with the core clinical services and infrastructure of a tertiary general hospital in place from the outset. The ICUs included in this study comprised the General ICU, Emergency ICU, and Paediatric ICU; non-ICU wards covered a full range of medical specialities, including internal medicine, surgery, gynaecology, obstetrics, paediatrics, and neonatology. Because 2018 was an incomplete operational year, the surveillance period comprised the first five complete calendar years after hospital opening, from 1 January 2019 to 31 December 2023. All procedures were approved by the ethics committee of the Seventh Affiliated Hospital, Sun Yat-sen University.

### 4.2. Bacterial Isolates and MDROs

Clinical bacterial isolates were collected from inpatients at the Seventh Affiliated Hospital, Sun Yat-sen University between January 2019 and December 2023. The target bacterial species under national antimicrobial resistance surveillance were *Klebsiella pneumoniae* (KP), *Escherichia coli* (ECO), *Acinetobacter baumannii* (AB), *Pseudomonas aeruginosa* (PA), *Staphylococcus aureus* (SA), *Enterococcus faecalis* (Efa), *and Enterococcus faecium* (Efm). The corresponding MDRO categories were CRKP, ESBL-KP, ESBL-ECO, CRECO, CRAB, CRPA, MRSA, VREfa and VREfm.

All isolates were recovered from specimens obtained during routine clinical care. No routine hospital-wide active surveillance programme for asymptomatic MDRO colonisation was conducted. Specimen types were classified as respiratory tract specimens; urine; blood; wound or pus specimens; abdominal drainage fluid, bile and other clinically relevant body fluids; other specimens; and unknown or unavailable. Respiratory tract specimens included sputum, endotracheal aspirates and bronchoalveolar lavage fluid. Other specimens included infrequently collected clinical specimens, such as tissue specimens, ear, nose and throat or ocular specimens, placental or fetal membrane tissue, gastrointestinal contents other than faeces, and other uncommon specimens.

To prevent duplicate isolates, only one isolate per bacterial species per patient per admission was retained, based on unique identification codes. Where multiple MDRO categories were identified during the same infection episode, each was retained separately for isolate-level and infection-episode analyses.

### 4.3. Microbiological Procedures

Bacterial identification was conducted using the VITEK MS automatic rapid microbial mass spectrometry system or the automated VITEK 2 system (bioMérieux, Marcy l’Etoile, France). Antimicrobial susceptibility testing was performed with the VITEK^®^ 2 (bioMérieux, Marcy l’Etoile, France), the Kirby–Bauer method, or the E-test method, in accordance with the manufacturer’s instructions. Zone diameters and minimum inhibitory concentration breakpoints were interpreted following the guidelines established by the Clinical and Laboratory Standards Institute (CLSI). ESBL production was assessed using the VITEK 2 antimicrobial susceptibility testing card and its Advanced Expert System (bioMérieux, Marcy l’Etoile, France). When the automated ESBL result was considered questionable or inconsistent with the overall susceptibility profile, phenotypic confirmation was performed using the combined-disk test in accordance with the laboratory protocol and applicable CLSI recommendations. A ≥5 mm increase in the inhibition zone diameter for a cephalosporin tested in combination with clavulanate, compared with the corresponding cephalosporin alone, was interpreted as confirmation of ESBL production. Molecular characterisation of carbapenemase-encoding genes (e.g., blaKPC, blaNDM, blaVIM, blaIMP, blaOXA-48-like) was not performed in this study, as routine laboratory protocols did not include genotypic screening for carbapenem resistance determinants. CRKP and CRECO were defined as isolates resistant to any carbapenem antimicrobial agent (imipenem, meropenem, or ertapenem); CRPA and CRAB were defined as isolates resistant to imipenem or meropenem. MRSA was defined as *S. aureus* resistant to oxacillin or cefoxitin. VREfa or VREfm were defined as *E. faecalis or E. faecium* resistant to vancomycin. Quality control strains—including *E. coli* ATCC 25922, *S. aureus* ATCC 25923 and ATCC 29213, *P. aeruginosa* ATCC 27853, and *E. faecalis* ATCC 29212—were obtained from the Clinical Testing Center of the National Health Commission.

### 4.4. IPC Measures and Compliance Monitoring

Standard precautions were implemented hospital-wide throughout the study period, while contact precautions were applied to patients with identified MDROs, as appropriate. MDRO-related infection prevention measures included isolation signage, appropriate use of personal protective equipment, dedicated or appropriately disinfected medical equipment, environmental cleaning and disinfection, healthcare waste management, and other MDRO-specific infection prevention measures where applicable [29]. Compliance with the selected measures listed in Table S5 was assessed through routine audits of a subset of MDRO cases. For each measure, annual compliance was calculated as the number of audited cases in which the measure was correctly implemented divided by the total number of audited cases assessed for that measure. Audit denominators varied by year and, where applicable, by measure.

### 4.5. Definitions and Classification of Infection and Colonisation

MDROs were categorised into two clinical states: infection and colonisation. An infection was characterised by the presence of symptomatic illness in patients, which is indicative of a pathogenic process involving the MDROs. Conversely, colonisation referred to the asymptomatic carriage of these organisms within the patient’s body, where they do not elicit a clinical response or disease. Infections and colonisation cases were diagnosed according to the Diagnostic Criteria for Nosocomial Infections (Trial) issued by the Ministry of Health of China [30]. Infections were further classified by onset: community-onset infections were defined as those present on admission or occurring within 48 h of admission, including both community-acquired cases and those transferred from other healthcare facilities; hospital-acquired infections were defined as those occurring more than 48 h after admission.

Infection sites were assigned on the basis of clinical diagnoses and infection-control surveillance records and were classified as lower respiratory tract infection or pneumonia, bloodstream infection, urinary tract infection, surgical-site infection, intra-abdominal infection, skin and soft-tissue infection, or other infection. Ventilator-associated pneumonia, catheter-associated urinary tract infection, and central line-associated bloodstream infection were recorded as device-associated subcategories when the relevant diagnostic criteria were met and were classified as hospital-acquired by definition.

### 4.6. Data Collection and Exclusion Criteria

**Figure 4:**
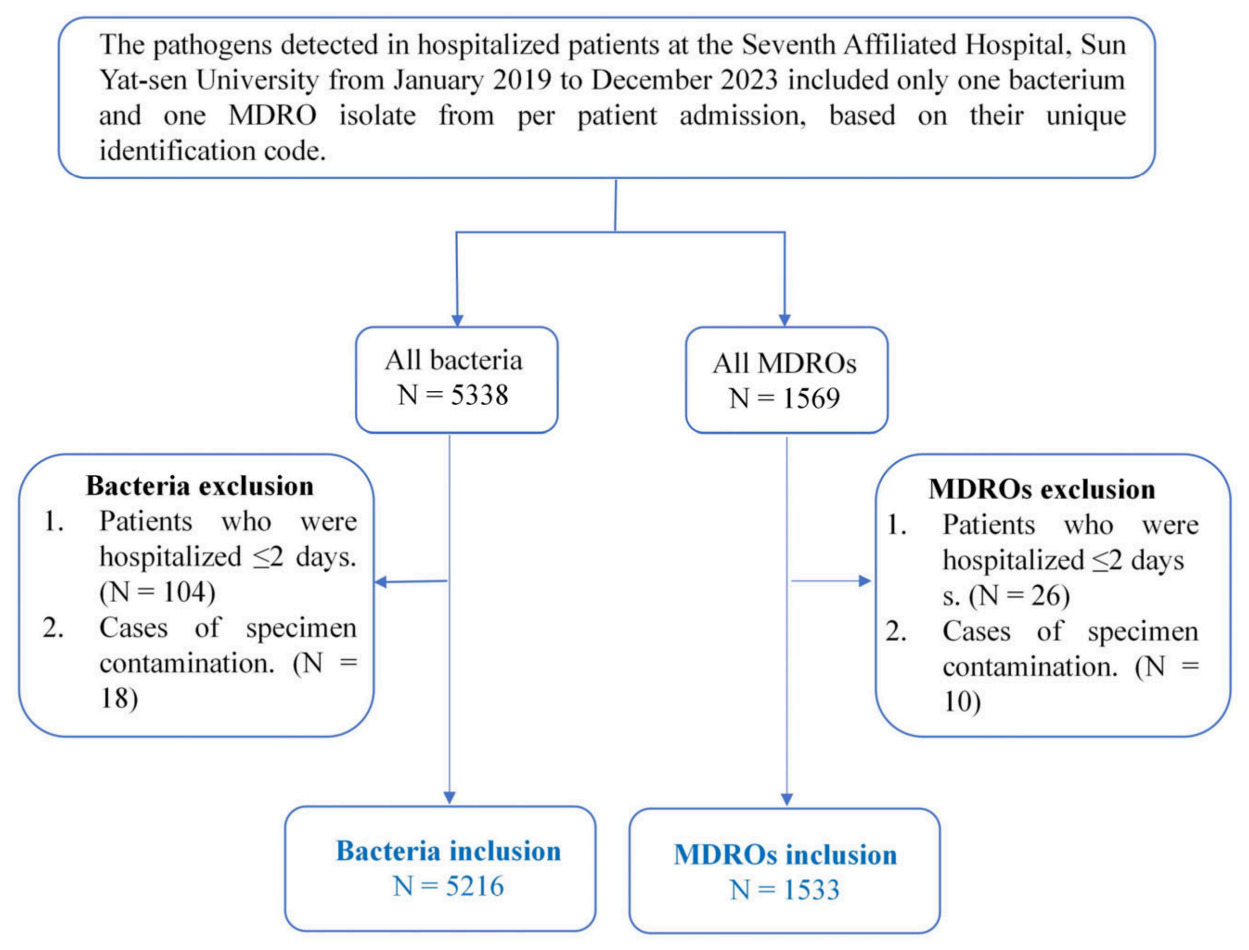
Flowchart of bacteria and MDROs exclusion and inclusion.

After excluding duplicate isolates, 5338 bacterial isolates and 1569 MDRO isolates were initially identified. We subsequently excluded: (i) patients hospitalised for <48 h (104 bacterial cases, 26 MDRO cases); and (ii) cases with confirmed specimen contamination (18 bacterial cases, 10 MDRO cases). A total of 5216 bacterial isolates and 1533 MDRO isolates were finally included in the analysis (Figure 4).

For the analysis of clinical characteristics and outcomes (Table S4), records were deduplicated using patient and admission identifiers within each infection-site category. Patients with infections at more than one site could contribute once to each applicable site-specific category but were counted only once in the overall patient total. Colonisation was excluded from both analyses. Clinical variables included ICU admission at any time during the index hospitalisation, in-hospital mortality, and total hospital length of stay. Data were extracted from electronic medical records and the IPC surveillance database.

### 4.7. External Benchmarking

Local MDRO isolation rates were compared with provincial surveillance data from the Guangdong Provincial Bacterial Resistance Monitoring Network [10] and national surveillance data from CHINET [9]. For national comparisons, isolation rates were benchmarked against the reported resistance rates to the corresponding antimicrobial classes (e.g., carbapenems for CRKP, CRAB, and CRPA; third-generation cephalosporins for ESBL-KP and ESBL-ECO). Although the local isolation rates and national resistance rates are derived from different populations (our single-centre clinical isolates versus multicentre surveillance data), this comparison was used to place local trends in the context of regional and national surveillance data. For MDROs without available provincial data, comparisons were made against national benchmarks only.

### 4.8. Antimicrobial Consumption Data

Annual hospital-wide antimicrobial consumption data for 2019–2023 were obtained from the hospital pharmacy department. Antimicrobial use intensity was expressed as defined daily doses (DDDs) per 100 bed-days and was calculated as follows:

Antimicrobial use intensity (DDDs per 100 bed-days) = (total DDDs of systemic antimicrobials consumed during the year/total number of bed-days during the year) × 100.

### 4.9. Statistical Analysis

MDRO isolation rate  (%) =  (number of isolates of a specific MDRO/total number of isolates of the same bacterial species in the same period)  ×  100.

MDRO hospital-acquired infections incidence density rate (/1000 patient days) =  (number of hospital-acquired infection cases of MDRO/total length of hospital stay in the same period)  ×  1000.

Pearson’s chi-square and Fisher’s exact tests were utilised based on the expected frequencies of categorical variables. Trend chi-square tests were conducted to analyse annual trends. Categorical variables were presented as frequencies and percentages, whereas continuous variables were presented as median (interquartile range, IQR). Statistical analyses were performed using Statistical Product and Service Solutions (SPSS), version 25.0 (IBM Corp, Armonk, NY, USA). Data visualisation was carried out with GraphPad Prism 8.0 (San Diego, CA, USA). *p* < 0.05 was considered statistically significant.

## Figures and Tables

**Table 1:** Comparison of MDRO isolation rates between ICU and Non-ICU wards.

MDROs		Total	ICU	Non-ICU	*p* *
ESBL-KP	ESBL-KP	252	40	212	0.088
	KP	997	195	802	
	Isolation rate (%)	25.3	20.5	26.4	
ESBL-ECO	ESBL-ECO	737	65	672	0.004
	ECO	1836	124	1712	
	Isolation rate (%)	40.1	52.4	39.3	
CRKP	CRKP	80	18	62	0.489
	KP	997	195	802	
	Isolation rate (%)	8.0	9.2	7.7	
CRECO	CRECO	51	11	40	<0.001
	ECO	1836	124	1712	
	Isolation rate (%)	2.8	8.9	2.3	
CRAB	CRAB	58	27	31	<0.001
	AB	312	84	228	
	Isolation rate (%)	18.6	32.1	13.6	
CRPA	CRPA	170	43	127	<0.001
	PA	616	97	519	
	Isolation rate (%)	27.6	44.3	24.5	
MRSA	MRSA	174	20	154	0.851
	SA	829	92	737	
	Isolation rate (%)	21.0	21.7	20.9	
VREfa	VREfa	3	0	3	0.672
	Efa	469	58	411	
	Isolation rate (%)	0.6	0	0.7	
VREfm	VREfm	8	0	8	0.278
	Efm	157	34	123	
	Isolation rate (%)	5.1	0	6.5	

*: ICU vs. Non-ICU ward. Abbreviations: ICU, Intensive Care Unit; ESBL, extended-spectrum β-lactamase-producing; MRSA, methicillin-resistant *Staphylococcus aureus;* CR, carbapenem-resistant; ECO, *Escherichia coli*; KP, *Klebsiella pneumoniae*; PA, *Pseudomonas aeruginosa*; AB, *Acinetobacter baumannii*; VREfa, vancomycin-resistant *Enterococcus faecalis*; VREfm, vancomycin-resistant *Enterococcus faecium*.

**Table 2:** Incidence density rate of MDRO infections in ICU and non-ICU in 2019–2023.

MDROs		Total	ICU	Non-ICU	*p* *
All MDROs	number of infections	554	96	458	
	Incidence density rate (/1000 patient days)	0.64	6.56	0.54	<0.001
ESBL-KP	number of infections	105	19	86	
	Incidence density rate (/1000 patient days)	0.12	1.30	0.10	0.011
ESBL-ECO	number of infections	254	30	224	
	Incidence density rate (/1000 patient days)	0.29	2.05	0.26	0.487
CRKP	number of infections	35	5	30	
	Incidence density rate (/1000 patient days)	0.04	0.34	0.04	0.648
CRECO	number of infections	17	1	16	
	Incidence density rate (/1000 patient days)	0.02	0.07	0.02	0.822
CRAB	number of infections	22	14	8	
	Incidence density rate (/1000 patient days)	0.03	0.96	0.01	<0.001
CRPA	number of infections	79	22	57	
	Incidence density rate (/1000 patient days)	0.09	1.50	0.07	<0.001
MRSA	number of infections	36	5	31	
	Incidence density rate (/1000 patient days)	0.04	0.34	0.04	0.694
VREfa	number of infections	1	0	1	
	Incidence density rate (/1000 patient days)	0	0	0	1.000
VREfm	number of infections	5	0	5	
	Incidence density rate (/1000 patient days)	0.01	0	0.01	1.000

*: ICU vs. Non-ICU. Abbreviations as in Table 1.

## Data Availability

The data presented in this study are available from the corresponding author upon reasonable request. The data are not publicly available because they contain information that could compromise patient confidentiality.

## Supplementary Materials

The following supporting information can be downloaded at: https://www.mdpi.com/article/10.3390/antibiotics15090834/s1, Table S1: Distribution of specimen types among included bacterial and MDRO isolates, 2019–2023, Table S2: Distribution of specimen types among the nine targeted MDROs, 2019–2023, Table S3: Distribution of primary infection sites among MDRO-associated infection records, stratified by onset classification, Table S4: Clinical characteristics and outcomes of patients with MDRO-associated infections, by infection site, Table S5: Annual compliance with selected MDRO-related infection prevention measures among routinely audited MDRO cases, 2019–2023.

## Abbreviations

The following abbreviations were used within the paper.

AMR	Antimicrobial resistance
ECO	*Escherichia coli*
MRSA	Methicillin-resistant *Staphylococcus aureus*
KP	*Klebsiella pneumoniae*
ESBL	Extended-spectrum β-lactamase
ESBL-ECO	ESBL-producing *Escherichia coli*
VREfm	Vancomycin-resistant *enterococcus faecium*
MDROs	Multidrug-resistant organisms
ICU	Intensive care unit
AB	*Acinetobacter baumannii*
PA	*Pseudomonas aeruginosa*
SA	*Staphylococcus aureus*
Efa	*Enterococcus faecalis*
Efm	*Enterococcus faecium*
CRKP	Carbapenem-resistant *Klebsiella pneumoniae*
ESBL-KP	ESBL-producing *Klebsiella pneumoniae*
CRECO	Carbapenem-resistant *Escherichia coli*
CRAB	Carbapenem-resistant *Acinetobacter baumannii*
CRPA	Carbapenem-resistant *Pseudomonas aeruginosa*
VREfa	Vancomycin-resistant *Enterococcus faecalis*
CLSI	Clinical and Laboratory Standards Institute
SPSS	Statistical Product and Service Solutions
IPC	Infection prevention and control
CRE	Carbapenem-resistant Enterobacteriaceae
