# Supplementary material for: Distinct Epidemiology and Temporal Trends of Multidrug-Resistant Organisms in a Newly Established Hospital: A Five-Year Surveillance Study
Source: Antibiotics (Basel). 2026 Aug 27;15(9):834. doi: 10.3390/antibiotics15090834 (PMC13603879; doi:10.3390/antibiotics15090834)
Supplement: Supplementary file 1 [file antibiotics-15-00834-s001.zip › antibiotics-4418071-supplementary.pdf]

Table S1. Distribution of specimen types among included bacterial and MDRO isolates, 2019–2023

Specimen type	All bacterial isolates, n	Proportion of all bacterial isolates, %	MDRO isolates, n	Proportion of MDRO isolates, %	MDRO proportion among target-species isolates, %
Respiratory tract specimens	1150	22.0	406	26.5	35.3
Urine	1630	31.2	486	31.7	29.8
Blood	439	8.4	136	8.9	31.0
Pus and wound specimens	308	5.9	122	8.0	39.6
Abdominal drainage fluid, bile, and other normally sterile body fluids	235	4.5	137	8.9	58.3
Other specimens	1390	26.6	246	16.0	17.7
Unknown/unavailable	64*	1.2	0	0	0
**Total**	**5216**	**100.0**	**1533**	**100.0**	**29.4**

* Specimen-type information was unavailable for 64 non-CRAB A. baumannii isolates. Data are presented as n or %. Abbreviations: MDRO, multidrug-resistant organism.

Table S2. Distribution of specimen types among the nine targeted MDROs, 2019–2023

Specimen type	ESBL-KP	ESBL-ECO	CRKP	CRECO	CRAB	CRPA	MRSA	VREfa	VREfm
Respiratory tract specimens	80 (31.7)	65 (8.8)	33 (41.2)	14 (27.5)	52 (89.7)	113 (66.5)	49 (28.2)	0 (0.0)	0 (0.0)
Urine	70 (27.8)	363 (49.3)	17 (21.2)	17 (33.3)	2 (3.4)	9 (5.3)	4 (2.3)	1 (33.3)	3 (37.5)
Blood	26 (10.3)	71 (9.6)	10 (12.5)	2 (3.9)	0 (0.0)	13 (7.6)	13 (7.5)	0 (0.0)	1 (12.5)
Pus and wound specimens	11 (4.4)	48 (6.5)	5 (6.2)	2 (3.9)	0 (0.0)	8 (4.7)	47 (27.0)	0 (0.0)	1 (12.5)
Abdominal drainage fluid, bile, and other normally sterile body fluids	23 (9.1)	80 (10.9)	11 (13.8)	9 (17.6)	2 (3.4)	5 (2.9)	4 (2.3)	2 (66.7)	1 (12.5)
Other specimens	42 (16.7)	110 (14.9)	4 (5.0)	7 (13.7)	2 (3.4)	22 (12.9)	57 (32.8)	0 (0.0)	2 (25.0)
Total	252 (100.0)	737 (100.0)	80 (100.0)	51 (100.0)	58 (100.0)	170 (100.0)	174 (100.0)	3 (100.0)	8 (100.0)

Data are presented as n (column %). Abbreviations: ESBL, extended-spectrum β-lactamase-producing; MRSA, methicillin-resistant *Staphylococcus aureus;* CR, carbapenem-resistant; ECO, *Escherichia coli*; KP, *Klebsiella pneumoniae*; PA, *Pseudomonas aeruginosa*; AB, *Acinetobacter baumannii*; VREfa, vancomycin-resistant *Enterococcus faecalis*; VREfm, vancomycin-resistant *Enterococcus faecium*.

Table S3. Distribution of primary infection sites among MDRO-associated infection records, stratified by onset classification

**Infection site/type**	**All MDRO-associated infection records (n = 1344)**	**Community-onset infections (n=790)**	**Hospital-acquired infections (n=554)**	***P* value**
Lower respiratory tract infection or pneumonia	371 (27.6)	204 (25.8)	167 (30.1)	0.081
Ventilator-associated pneumonia†	14 (1.0)	0 (0.0)	14 (2.5)	—
Bloodstream infection	136 (10.1)	49 (6.2)	87 (15.7)	<0.001
Central line-associated bloodstream infection†	56 (4.2)	0 (0.0)	56 (10.1)	—
Urinary tract infection	431 (32.1)	298 (37.7)	133 (24.0)	<0.001
Catheter-associated urinary tract infection†	48 (3.6)	0 (0.0)	48 (8.7)	—
Surgical-site infection	55 (4.1)	12 (1.5)	43 (7.8)	<0.001
Intra-abdominal infection	150 (11.2)	79 (10.0)	71 (12.8)	0.107
Skin and soft-tissue infection	88 (6.5)	68 (8.6)	20 (3.6)	<0.001
Other infections	113 (8.4)	80 (10.1)	33 (6.0)	0.007
**Total**	**1344 (100.0)**	**790 (100.0)**	**554 (100.0)**	—

The unit of analysis was the MDRO category-specific infection record. Different MDRO categories identified during the same clinical infection episode were retained as separate records. † Device-associated infection rows are subcategories included within the corresponding lower respiratory tract, bloodstream, or urinary tract infection rows; consequently, the displayed row percentages are not intended to sum to 100%. Data are presented as n (column %). *P* values compare community-onset and hospital-acquired infections for each parent infection-site row.

Table S4. Clinical characteristics and outcomes of patients with MDRO-associated infections, by infection site

Infection site/type	No. of patients	In-hospital mortality n (%)	ICU admission during index hospitalisation, n (%)	Length of hospital stay, median (IQR), days
Lower respiratory tract infection or pneumonia	319	45 (14.1)	150 (47.0)	28 (15–50.5)
Ventilator-associated pneumonia†	14	6 (42.9)	13 (92.9)	61 (28.8–127)
Bloodstream infection	125	13 (10.4)	32 (25.6)	30 (17–56)
Central line-associated bloodstream infection†	53	10 (18.9)	16 (30.2)	43 (29–73)
Urinary tract infection	416	11 (2.6)	54 (13.0)	15 (8–24)
Catheter-associated urinary tract infection†	45	1 (2.2)	14 (31.1)	22 (15–50)
Surgical-site infection	49	0 (0.0)	12 (24.5)	33 (23–51)
Intra-abdominal infection	133	8 (6.0)	41 (30.8)	24 (15–40)
Skin and soft-tissue infection	81	0 (0.0)	13 (16.0)	16 (8–31)
Other infections	110	6 (5.5)	13 (11.8)	14 (6–29.5)
**Total**	**1196**	**77 (6.4)**	**297 (24.8)**	**20 (10–37)**

Data are presented as n (%) or median (IQR). Patients with infections at more than one site could contribute once to each applicable site-specific category but were counted only once in the overall total. †Device-associated infection rows are subcategories included within their corresponding parent infection-site rows. ICU admission refers to admission at any time during the index hospitalisation. Abbreviations: ICU, intensive care unit; IQR, interquartile range.

Table S5. Annual compliance with selected MDRO-related infection prevention measures among routinely audited MDRO cases, 2019–2023

MDRO-related infection prevention measure	2019, n/N (%)	2020, n/N (%)	2021, n/N (%)	2022, n/N (%)	2023, n/N (%)
Isolation signage	113/123 (91.9)	115/127 (90.6)	220/228 (96.5)	157/161 (97.5)	237/254 (93.3)
Appropriate use of personal protective equipment	121/123 (98.4)	125/127 (98.4)	224/228 (98.2)	143/161 (88.8)	217/254 (85.4)
Dedicated or appropriately disinfected medical equipment	94/122 (77.0)	122/127 (96.1)	220/228 (96.5)	159/161 (98.8)	222/254 (87.4)
Environmental cleaning and disinfection	102/123 (82.9)	122/127 (96.1)	225/228 (98.7)	157/161 (97.5)	248/254 (97.6)
Healthcare waste management	68/123 (55.3)	111/127 (87.4)	210/228 (92.1)	152/161 (94.4)	187/254 (73.6)

Data are presented as n/N (%), where n is the number of audited MDRO cases in which the specified measure was correctly implemented and N is the number of audited cases assessed for that measure. Data were obtained from routine audits of a subset of MDRO cases rather than complete assessment of all MDRO cases. Abbreviations: MDRO, multidrug-resistant organism.
